# Supplementary figures and images for: Predictive factors for long-term survival after surgery for pancreatic ductal adenocarcinoma: Making a case for standardized reporting of the resection margin using certified cancer center data
Source: PLoS One. 2021 Mar 18;16(3):e0248633. doi: 10.1371/journal.pone.0248633 (PMC7971889; doi:10.1371/journal.pone.0248633)

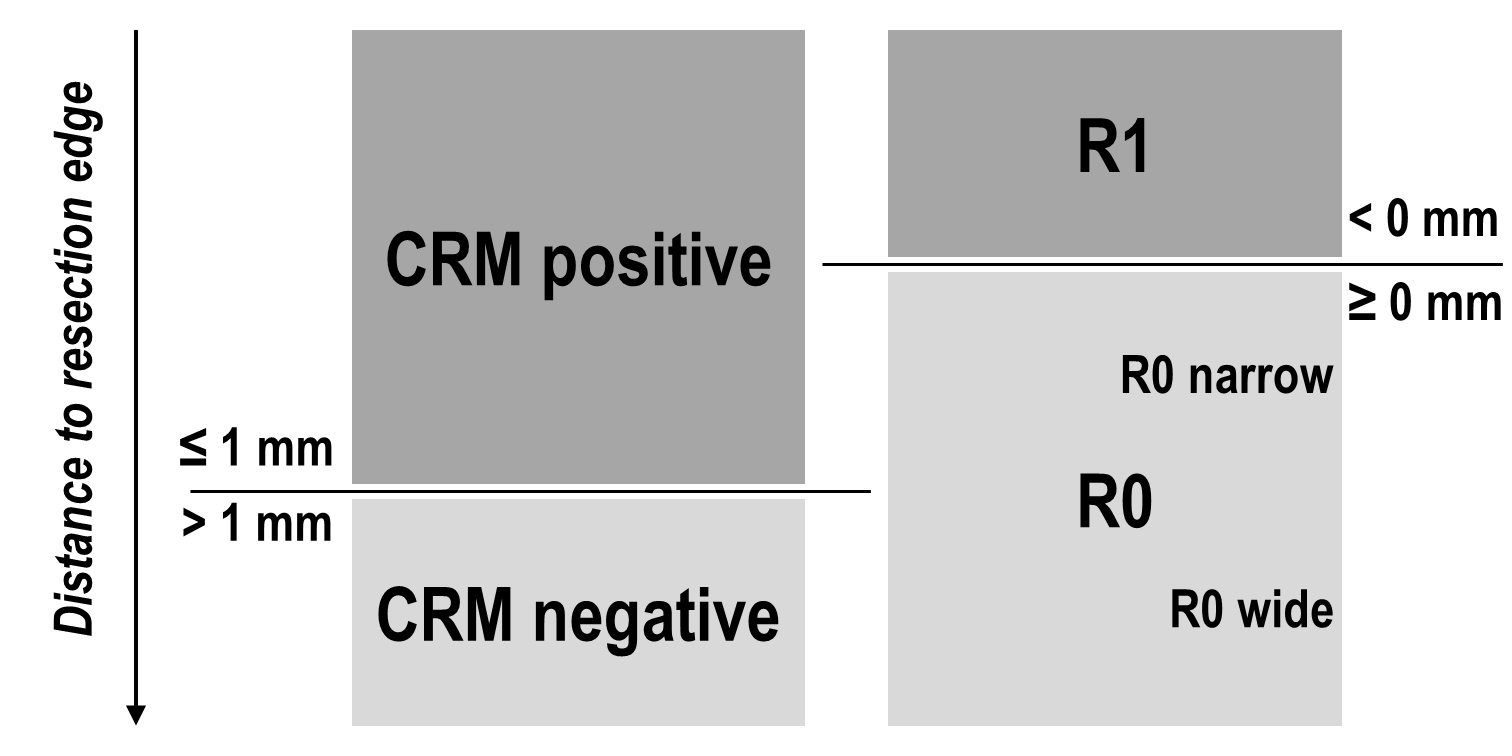

Supplement: S1 Fig — (TIF) [file pone.0248633.s001.tif]
