# Supplementary material for: Predictive factors for long-term survival after surgery for pancreatic ductal adenocarcinoma: Making a case for standardized reporting of the resection margin using certified cancer center data
Source: PLoS One. 2021 Mar 18;16(3):e0248633. doi: 10.1371/journal.pone.0248633 (PMC7971889; doi:10.1371/journal.pone.0248633)
Supplement: S1 File — (PDF) [file pone.0248633.s002.pdf]

Oncol Res Treat
41(suppl 1) VII–221 (2018)

41 | S1 | 18

print
ISSN 2296–5270
e-ISBN 978-3-318-04837-7

online
e-ISSN 2296–5262

www.karger.com/ort

Oncology Research and Treatment

Band 41,
Supplement 1,
Februar 2018
online only

33. Deutscher Krebskongress

Perspektiven verändern Krebs – Krebs verändert Perspektiven

Diagnose – Therapie – (Über-)Leben

ABSTRACTS

Berlin, 21.–24. Februar 2018

Herausgeber
Thomas Wiegel, Ulm

33. DEUTSCHER KREBSKONGRESS 2018

S. Karger
Medical and Scientific Publishers
Basel · Freiburg · Paris · London ·
New York · Chennai · New Delhi ·
Bangkok · Beijing · Shanghai ·
Tokyo · Kuala Lumpur ·
Singapore · Sydney

KARGER

reduced after full skin transplantation, but the range of movement was still within the normal range for all patients. All patients were satisfied with both functional and aesthetic results.
Conclusions: Both procedures for the closure of the radial forearm donor site lead to good functional as well as aesthetic results. The wound healing of the split thickness skin removal defect is significantly longer, whereas in patients with full-skin transplants, a functionally rarely limited reduction of mobility in the wrist is observed.

References:

1. Ho T, Couch M, Carson K, Schimberg A, Manley K, Byrne PJ: Radial forearm free flap donor site outcomes comparison by closure methods. Otolaryngol Head Neck Surg. 2006;134(2):309–315.

Disclosure Statement: The authors declare that there is no conflict of interest.

672

Circumferential Resection Margin influences long term survival after pancreatic adenocarcinoma

D. Weyhe[1]; V. Uslar[1]; B. Sahlmann[1]; A. Tannapfel[2]

[1]Universität Oldenburg, Universitätsklinik für Viszeralchirurgie, Medizinischer Campus Universität Oldenburg, Pius-Hospital, Oldenburg, Deutschland
[2]Ruhr-Universität Bochum, Institut für Pathologie, Institut für Pathologie, Bochum, Deutschland

Purpose: Leading factor for prognosis after pancreatic adenocarcinoma is, together with tumor stage, the nodal status and a free resection margin. Histopathology reports use R-status inconsistently. Therefore, R-status is seen controversially. It is unclear, if the additional classification of Circumferential Resection Margin (CRM) is a better predictor.
Methods: Between January 2010 and June 2017 our certified pancreas center treated n = 333 pat. For pancreatic cancer (n = 154 receiving pancreatic resection). Only pat with ductal adeno carcinoma were included (n = 121; mean age = 68yrs). Histology was performed by standardized protocol. R0 was supplemented by CRM > 1mm (negative) and CRM≤1mm (positive). Tumor documentation was carried out prospectively in accordance to the established information system with a follow up rate of >90%. Dependent variables were UICC status, R-status, lymph node ratio (LNR), and CRM. Influence on overall survival (Kaplan-Meier), backward stepwise Cox regression, and likelihood of survival >24mo (Odds ratio) was evaluated.
Results: No differences were found for survival if stratified by R-status. CRM significantly discriminates for long term survival. Only 7.2% of all CRM positive pat survived longer than 24mo after diagnosis, as compared to 28.9% of CRM negative pat (OR: 5.23, p = 0.003). Cox regression showed that overall survival depends on UICC and CRM status ($Chi^2 = 14.2$, p = 0.027).
Conclusions: CRM status leads -together with UICC- to a better prediction than R-status alone. CRM status therefore should be part of histopathology after standardized preparation of the resected material, since CRM status seems to be an important factor influencing long term survival.

Disclosure Statement: All authors have nothing to disclose.

741

Long-term metabolic changes after cancer-related pancreatic resection – first results of an RCT study

M. Hoffmann[1]; B. Sahlmann[1]; V. Uslar[1]; N. Tabriz[1]; M. Droste[2]; D. Weyhe[1]

[1]Universität Oldenburg, Universitätsklinik für Viszeralchirurgie, Medizinischer Campus Universität Oldenburg, Pius-Hospital, Oldenburg, Deutschland
[2]Medicover Mvz Oldenburg, Oldenburg, Deutschland

Purpose: Inadequate nutrition and metabolic irregularities can be a companion for postoperative pancreatic surgery and have a far-reaching impact on postoperative general health (GH) and quality of life (QoL). Therefore, the pPRP study (DRKS study no: DRKS00006786) was conceived to monitor GH and QoL after pancreatic resection over the course of 1 year depending on the type of physiotherapy patients receive.
Methods: The RCT is designed as a pilot study (n = 60 patients), in which QoL and GH are evaluated by means of a structured follow-up. One group of patients is given intensive physiotherapy (IP; include use of a bed bicycle beginning directly after extubation) and the other group standard physiotherapy (SP) after tumor-related pancreatic resection. Follow ups include monthly telephone calls, n = 3 postop ECG and CT measurements, and metabolic analysis of 25 different metabolic parameters to reveal potential deficiency symptoms.
Results: Currently, n = 45 patients are included. All IP patients perceive the early postoperative movement therapy (bed bicycle) very positively. Magnesium (median: 30mg/l; norm: 30–40), selenium (70µg/l; 65–150), Vitamin D (19.7ng/ml; >20), Vitamin B1 (31 µg/l,> 38), and B6 (5.2 µg/l;> 9), as well as Hb (11.4 g/dl; 12–15.4) are at the lower limit or even below the standard range for SP and IP. No significant differences between IP and SP are observed.
Conclusions: Important metabolic parameters are significantly below the standard after pancreatic resection independent of the amount of physical activity. Whether and how missing metabolic substances should be substituted has to be clarified in further studies.

Disclosure Statement: All authors have nothing to disclose.

787

Intravascular (post-hyterectomy) leiomyoma (IVL) as late Tumor Thrombus within the inferior vena cava (IVC) – a rare case primarily imposing as IVC thrombus´originating from left renal vein after former left nephrectomy Status (case report & review of literature)

M. Ghanem[1]; F. Meyer[2]; V. Schoeder[3]; A. Ignatov[4]; M. Fadel[5]; Z. Halloul[6]

[1]University Hospital at Magdeburg, Division of Vascular Surgery, Dept. of General, Abdominal, Vascular & Transplant Surgery, Magdeburg, Deutschland
[2]Universitätsklinikum Magdeburg A.Ö.R., Gefäß- und Schlaganfallzentrum, Arbeitsbereich Gefäßchirurgie, Klinik für Allgemein-, Viszeral- & Gefäßchirurgie, Magdeburg, Deutschland
[3]University Hospital at Magdeburg, Inst. of Pathology, Magdeburg, Deutschland
[4]University Hospital at Magdeburg, Dept. of Gynecology & Obstetrics, Magdeburg, Deutschland
[5]Mohammad, Fadel, Magdeburg, Deutschland
[6]Uniklinik Magdeburg, Magdeburg, Deutschland

Introduction: Intravascular leiomyoma is a rare type of myoma. It was firstly described by Birch-Hirschfeld in 1896, however, its intracardiac subtype was firstly reported by Durck in 1907. Most patients are asymptomatic. The tumor invades mostly the tributaries of the inferior vena cava (IVC) with upward extension that may approach the intracardiac space.
Aim & Method: By means of a scientific case report, a patient with the very rare diagnosis of an endocaval leiomyoma thrombus post hysterectomy is described based on the clinical experiences obtained in the specific case management and selective references from the literature.
Case presentation: A 48-years old female was diagnosed with intravascular tumor growth within the IVC with intracardiac extension using chest and abdominal CT scan, ECG and echocardiography which was approached by an interdisciplinary (vascular and cardiothoracic) surgical intervention including heart-lung machine with favorable postoperative result (R0 resection status with mid-term outcome, no recurrent tumor growth). Histopathological investigation diagnosed leiomyoma origin already from ovarian vein most likely in context to the former hysterectomy (3 years ago).
Discussion & conclusion: Intravascular leiomyoma is a benign tumor with invasive tendency, which can be considered a diagnostic and therapeutic challenge. It should be thoroughly investigated to be planned for a radical surgical removal. By possible adherence to the intraabdominal or -thoracic organs, an interdisciplinary and eventually step-wise surgical approach (combining vascular, abdominal, thoracic and heart surgery as well as gynecology and urology), which can be demanding, is recom-
